# Reducing cognitive load in dispatcher-assisted CPR: a simulation-based educational study among non-medical university students

**DOI:** 10.1186/s12245-026-01128-x

**Published:** 2026-02-02

**Authors:** Nantawan Tippayanate, Phacharee Phonkanya, Kanokwan Nuangkantee, Kamonchanok Nuangkantee, Aphisit Moolsombat, Chattarin Sripol, Souksathaphone Chanthamath

**Affiliations:** 1 Faculty of Medicine, Mahasarakham University, Mahasarakham, 44000 Thailand; 2 Command Control Center, Subdistrict Administrative Organization, Mahasarakham, 44000 Thailand; 3 Emergency Department, Mahasarakham Hospital, Mahasarakham, 44000 Thailand; 4 Khammouane Provincial Hospital, Thakhek, Lao PDR; 5 International and National Collaborative Network and Innovation for Community Health Development Research Unit (INCHDI), Mahasarakham University, Mahasarakham, 44000 Thailand

**Keywords:** Dispatcher-assisted CPR, Cognitive load, Simulation-based education, Emergency medical dispatcher, Instructional design

## Abstract

**Background:**

Dispatcher-assisted cardiopulmonary resuscitation (DA-CPR) is critical for improving outcomes after out-of-hospital cardiac arrest, yet untrained callers often experience high cognitive load that impedes early recognition and action. Communication strategies grounded in instructional design may reduce this burden. We evaluated whether a simplified dispatcher script developed using Cognitive Load Theory (CLT) and the ADDIE framework improves DA-CPR performance in a simulated setting.

**Methods:**

We conducted a simulation-based randomized study among undergraduate non-medical students in Thailand. Participants without prior CPR training were randomly assigned to receive either the standard NIEMS 2013 dispatcher script or a CLT–ADDIE simplified script during a simulated cardiac arrest scenario. Participants were blinded to allocation; dispatchers received concealed assignment prior to simulation, and outcome assessors were blinded. Primary outcomes were time to cardiac arrest recognition and time to first chest compression. Secondary outcomes included CPR quality and perceived cognitive load measured by the 9-point Paas scale.

**Results:**

Forty-two participants completed the simulation. Cardiac arrest recognition within 60 s was more frequent in the simplified group than in the standard group (90.5% vs. 47.6%, p = 0.034). Median recognition time was shorter with the simplified script (52 [45–60] vs. 74 [60–90] seconds), and perceived cognitive load was lower (median 4 [IQR 3–5] vs. 6 [IQR 5–7], p = 0.018). Qualitative observations suggested clearer task sequencing and reduced confusion.

**Conclusion:**

A CLT–ADDIE–based simplified dispatcher script improved early recognition of cardiac arrest and reduced cognitive load in a simulated DA-CPR context. Instructional design–informed dispatcher communication may enhance bystander performance during time-critical emergencies.

## Introduction

Out-of-hospital cardiac arrest (OHCA) remains a leading cause of sudden death worldwide, and survival depends heavily on early recognition, rapid chest compression, and effective dispatcher-assisted cardiopulmonary resuscitation (DA-CPR) [1, 2]. Dispatchers represent the first operational link in the chain of survival, but the clarity and sequencing of their verbal instructions strongly influence bystander willingness and performance [3].

Recent European and Scandinavian studies have shown that differences in dispatcher questioning, speech tempo, and caller engagement significantly affect the likelihood and timeliness of bystander CPR [4–6]. Hardeland et al. demonstrated marked variation in cardiac arrest recognition across Nordic dispatch centers (71–96%), linked to communication structure and the handling of abnormal-breathing questions [4]. Likewise, Eberhard et al. emphasized that standardized dispatcher support is one of the most cost-effective ways to improve neurologically intact survival [5]. Nevertheless, excessive detail or fragmented language can increase caller hesitation and prolong time to compression [6].

Moreover, qualitative and mixed-methods studies have shown that emotional stress, fear of failure, and uncertainty under pressure further reduce cognitive readiness and decision-making capacity among untrained callers during dispatcher-assisted CPR respectively [7–8]. These findings support the notion that communication strategies should minimize emotional and cognitive overload to promote timely and confident action by lay rescuers.

In Thailand, the National Institute for Emergency Medicine (NIEM) established a standardized DA-CPR protocol in 2013 to guide emergency medical dispatchers (EMDs) [9]. Although comprehensive, this script often contains redundant or complex phrasing that imposes high cognitive demand on callers with limited working memory. Under time pressure and stress, untrained lay rescuers experience increased intrinsic load, which can delay comprehension and response. This phenomenon aligns with findings from recent qualitative work showing that emotional stress, fear of failure, and lack of confidence reduce cognitive capacity and decision readiness during real or simulated cardiac arrest events [10].

Cognitive Load Theory (CLT) distinguishes between intrinsic, extraneous, and germane load, emphasizing that learning and task performance improve when extraneous processing is minimized and essential elements are prioritized [10–11]. Applying CLT to dispatcher communication allows simplification of verbal guidance to reduce unnecessary cognitive load while maintaining task fidelity. The ADDIE instructional design framework offers a structured process for developing and refining such interventions; integrating CLT into ADDIE enables systematic optimization of message clarity, sequencing, and motivational cueing [12].

Therefore, this study aimed to evaluate whether a simplified DA-CPR script developed using CLT and ADDIE principles could improve time-critical performance and reduce perceived cognitive load among untrained laypersons in simulated dispatcher-assisted cardiac arrest scenarios.

## Methods

To improve clarity and reproducibility, the full NIEM 2013 dispatcher script and the CLT-informed simplified script are presented below in complete script format. Each step is shown verbatim to illustrate structural and cognitive differences. In the simplified script, address verification was intentionally omitted to isolate CPR instruction performance, consistent with international variability in DA-CPR protocols and dispatcher workflow models that allow task prioritization.

### Study design and setting

This was a simulation-based randomized comparative study, conducted at the Faculty of Medicine, Mahasarakham University, Thailand. This study followed the CONSORT 2010 extension for randomized pilot and simulation trials. The study received ethical approval (approval no. 208–238/2565). Participants were randomly assigned to either the standard or simplified DA-CPR script groups using a computer-generated sequence. The simulation sessions were held in four identical rooms equipped with adult CPR manikins and video recording systems.

Effect sizes were calculated for between-group comparisons to estimate practical significance.

Rank-biserial correlation (r) was reported for Mann–Whitney U tests, and Cramer’s V for Chi-square tests, interpreted according to Cohen’s conventional thresholds (small = 0.1, medium = 0.3, large = 0.5).

Participants were randomly assigned to either the standard or simplified DA-CPR script groups using a computer-generated sequence. Group allocation was concealed using sequentially numbered opaque envelopes prepared by an independent researcher not involved in data collection or outcome assessment. Participants and outcome assessors were blinded to group allocation. Due to the nature of the verbal script–based intervention, dispatchers could not be blinded during script delivery; however, they were unaware of group assignment until entering the simulation room.

### Participants

Forty-two undergraduate students from non-health science programs participated in the study. None had prior CPR training. All participants provided written informed consent and were blinded to the study hypothesis.

### Intervention: standard vs. simplified DA-CPR scripts

In the simplified script, address verification was intentionally omitted because the simulation focused exclusively on evaluating CPR instruction performance. This design choice is consistent with international evidence showing substantial heterogeneity in DA-CPR workflows, where many systems adapt, shorten, or omit preliminary steps depending on local dispatch strategies and operational priorities [13]. This approach aligns with recognized EMD workflow models, which allow task prioritization such as shifting directly to CPR-first instructions in selected educational or simulation settings [14].

The control group followed the NIEMS 2013 dispatcher script containing 21 sequential steps [9].

The intervention group received a simplified script of 16 concise, single-action commands developed through the ADDIE process guided by CLT. Key design features included:

1. Segmenting: each prompt contained one action only.
2. Signaling: key terms were emphasized to direct attention.
3. Redundancy reduction: unnecessary explanations were removed.
4. Motivational cueing: brief encouragement was added (“You can save this life—start now!”).

All dispatchers were trained for standardized tone, pacing, and delivery.

**Fig. 1:**
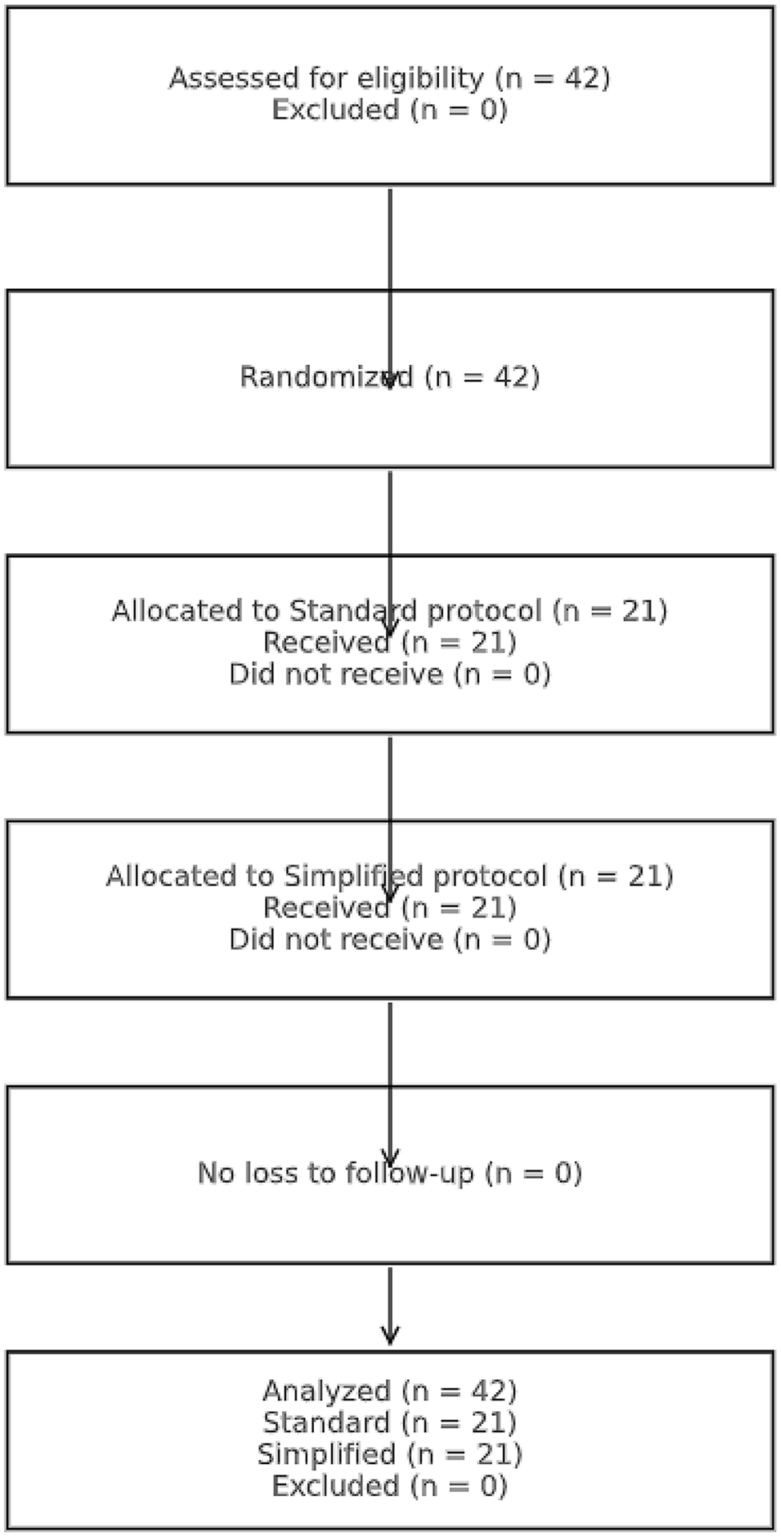
Participant flow diagram comparing the standard and simplified dispatcher-assisted CPR (DA-CPR) scripts

A comparison of the standard NIEMS 2013 dispatcher script and the simplified CLT–ADDIE version is presented in Fig. 1.

### Data collection and measures

A medium-fidelity simulation was conducted to replicate dispatcher-assisted CPR scenarios using a feedback-capable manikin that recorded compression depth, rate, and recoil (Resusci Anne, Laerdal, Norway). Each participant completed one simulated emergency call, guided by a dispatcher following either the standard or simplified protocol. Video and manikin data were analyzed by two blinded assessors.

Primary outcomes included time to cardiac arrest recognition (≤ 60 s) and time to first chest compression (≤ 4 min). The thresholds of ≤ 60 s and ≤ 4 min were predefined performance targets based on prior DA-CPR literature and were not derived from study results.

Cognitive load was quantified using the Paas 9-point Mental Effort Rating Scale, a validated single-item measure widely used in cognitive load research. After completing each scenario, participants rated the mental effort required to follow dispatcher instructions on a scale from 1 (‘very, very low mental effort’) to 9 (‘very, very high mental effort’). Higher scores indicated greater perceived cognitive load. This scale has been used extensively to evaluate instructional design efficiency and real-time task demands.

Secondary outcomes included correct hand placement, full chest recoil, compression depth (≥ 5 cm), and compression rate (100–120 bpm).

After each scenario, perceived mental effort was explored qualitatively during post-simulation interviews, asking participants to describe the difficulty and stress level of performing CPR under dispatcher guidance; higher ratings indicated greater mental effort.

### Statistical analysis

Statistical analyses were performed using IBM SPSS Statistics version 29. Descriptive data are presented as mean ± standard deviation (SD) or number (percentage), as appropriate.

Normality of continuous variables was assessed using the Shapiro–Wilk test and visual inspection of histograms. Several primary outcome variables demonstrated non-normal distributions; therefore, non-parametric tests were applied. Time intervals and cognitive load scores were expressed as median [IQR] and compared between groups using the Mann–Whitney U test. Effect sizes were calculated using rank-biserial correlation (r) to support interpretation of practical significance.

Categorical variables were presented as n (%) and analyzed using Chi-square tests, with Cramer’s V reported as the effect size where appropriate. Statistical significance was defined as *p* < 0.05.

Self-rated cognitive effort was assessed using the 9-point Paas scale (1 = very low, 9 = very high) [15]. Qualitative data from observer field notes and post-simulation interviews were analyzed using an inductive thematic approach. Two researchers independently coded the data, compared coding frameworks, and resolved discrepancies through discussion to ensure analytic rigor. Themes were generated to capture recurring perceptions related to task difficulty, attentional demands, emotional stress, and overall cognitive load.

## Results

**Fig. 2:**
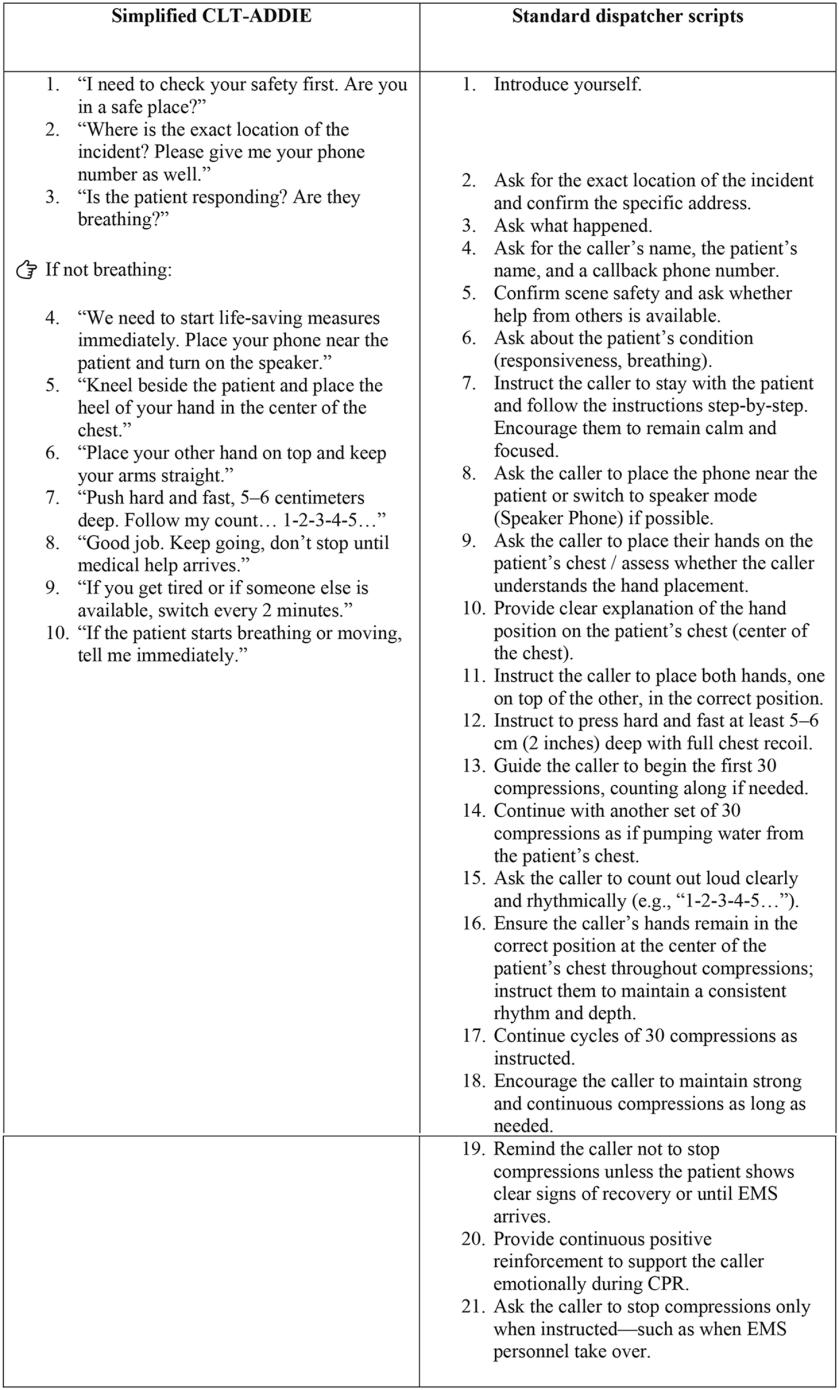
CONSORT flow diagram showing participant enrollment, randomization, allocation, and analysis

Participant enrollment, allocation, and analysis are summarized in the CONSORT flow diagram (Fig. 2).

## Participant characteristics

All 42 participants completed the simulation. Groups were comparable at baseline (Table 1): mean age 20.5 ± 1.2 years and 50.0% female overall, with similar distributions of fields of study. No participant reported prior CPR or emergency training.

**Table 1:** Baseline characteristics of participants

Characteristic	Standard protocol (*n* = 21)	Simplified protocol (*n* = 21)	Total (*n* = 42)
Age (years), mean ± SD	20.6 ± 1.3	20.4 ± 1.2	20.5 ± 1.2
Female, n (%)	11 (52.4%)	10 (47.6%)	21 (50.0%)
Field of study, n (%)			
Engineering	9 (42.9%)	8 (38.1%)	17 (40.5%)
Education	7 (33.3%)	8 (38.1%)	15 (35.7%)
Humanities & others	5 (23.8%)	5 (23.8%)	10 (23.8%)
Prior CPR or first-aid training, n (%)	0 (0%)	0 (0%)	0 (0%)
CPR experience (simulated only), n (%)	0 (0%)	0 (0%)	0 (0%)
Dispatcher exposure (EMS familiarity), n (%)	0 (0%)	0 (0%)	0 (0%)

Note: Data are mean ± SD or n (%). Groups were randomized; therefore, in accordance with CONSORT2010 and SJTREM policy, baseline characteristics were described without formal hypothesis testing

Abbreviations: SD, standard deviation; EMS, emergency medical services

### Primary outcomes

Recognition of cardiac arrest within 60 seconds was achieved more frequently in the simplified-protocol group than in the standard group (90.5% vs. 47.6%, *p* = 0.034) (Table 2).

The median time to cardiac arrest recognition was 52 [45–60] seconds in the simplified group, compared with 74 [60–90] seconds in the standard group.

The median time to first chest compression was shorter in the simplified group (188 [160–220] seconds vs. 260 [200–300] seconds), although this difference was not statistically significant (*p* = 0.217).

**Table 2:** Comparison of time-critical performance outcomes

Variable	Standard protocol	Simplified protocol	*p*-value	Effect size (*r*)
Time to recognition (s), median [IQR]	74 [60–90]	52 [45–60]	0.034	0.38
Time to first compression (s), median [IQR]	260 [200–300]	188 [160–220]	0.217	0.22

Data are presented as median [IQR] or n (%). Between-group comparisons were performed using the Mann–Whitney U and Chi-square tests

### Secondary outcomes

Self-reported Paas 9-point scores indicated lower perceived mental effort in the simplified group (median = 4 [IQR 3–5]) compared with the standard group (median = 6 [IQR 5–7], *p* = 0.018). Participants described the simplified protocol as “easier to follow” and “less stressful,” reflecting reduced cognitive load associated with clearer, single-action prompts and motivational phrasing.

**Table 3:** Comparison of CPR quality metrics and cognitive load between groups

Performance metric	Standard protocol	Simplified protocol	*p*-value	Effect size
Depth (cm)	21 (< 5 cm), 0 (≥ 5 cm)	21 (< 5 cm), 0 (≥ 5 cm)	0.739	V = 0.06
Rate (beats/min)	< 100: 17, 100–120: 2, > 120: 2	< 100: 20, 100–120: 1, > 120: 0	0.316	V = 0.15
Full recoil, n (%)	4 (19.0)	9 (42.9)	0.177	V = 0.23
Cognitive load (median [IQR])	6 [5–7]	4 [3–5]	0.018	*r* = 0.37

Data are presented as n (%) or median [IQR]

Between-group comparisons were performed using Chi-square tests for categorical variables and Mann–Whitney U tests for continuous or ordinal variables

Effect sizes were calculated as Cramer’s V (for categorical data) and rank-biserial correlation r (for continuous data), interpreted according to Cohen’s conventions (small = 0.1, medium = 0.3, large = 0.5)

*p* < 0.05 was considered statistically significant

The comparison of CPR performance variables between the simplified and standard protocol groups is presented in Table 3. The simplified protocol yielded a moderate effect size for reduced cognitive load (*r* = 0.37) and a small-to-moderate effect for improved chest recoil (V = 0.23), supporting the practical relevance of the observed differences despite limited statistical power.

### Qualitative observations

Video and field-note analyses revealed consistent behavioral themes among participants during dispatcher-assisted CPR simulations.

First, hesitation and overreliance on dispatcher prompts were common, as participants frequently paused or waited for explicit confirmation before acting. When dispatcher guidance stopped momentarily, most participants also ceased compressions, indicating strong dependence on external verbal cues.

Second, posture and hand-placement errors were frequent; many knelt too close to the manikin or positioned their hands too low on the chest with flexed fingers, resulting in insufficient depth (< 5 cm).

Third, difficulty estimating compression depth and maintaining rhythm reflected limited spatial and kinesthetic awareness among untrained rescuers.

These patterns illustrate high intrinsic and extraneous cognitive load under time pressure, even in a calm simulation environment. The findings support the quantitative results—showing that simplified, concise, and motivational dispatcher cues can help reduce cognitive burden and sustain bystander performance.

The comparative thematic analysis between the standard and simplified DA-CPR protocols further highlighted efficiency gains. The simplified script shortened overall call duration approximately 5–6 min versus 7–8 min, initiated chest compressions earlier, and reduced redundant verification steps. Earlier introduction of motivational phrases improved engagement, while omitting unnecessary safety inquiries enhanced flow and confidence. Overall, concise and prioritized communication contributed to faster action, greater continuity, and reduced cognitive demand among novice rescuers.

### Key difference

The simplified script eliminates verification and background steps and prioritizes rapid assessment and immediate action, especially starting compressions earlier.

## Discussion

Recent real-world evidence reinforces the importance of structured, cognitively efficient dispatcher communication. Analysis of EMS call-centre performance in systems lacking standardized questioning or DA-CPR protocols has shown delayed recognition of cardiac arrest, incomplete assessment of abnormal breathing, and extremely low rates of telephone-assisted CPR—often below 5% [16]. These operational gaps have been linked to prolonged reaction times, reduced bystander CPR, and a lower proportion of shockable rhythms upon EMS arrival [16]. Such findings mirror the cognitive and emotional barriers identified in our qualitative themes, where callers described confusion with multi-step commands, difficulties maintaining attention, and stress that interfered with action [7–9]. Viewed alongside our simulation results, this external evidence strengthens the rationale for CLT-informed simplification: reducing extraneous load and providing clear, concise instructions directly addresses known bottlenecks in the dispatch-to-caller interaction [13–15].

The recognition and initiation times measured in this study represent the caller-side response to dispatcher instructions, corresponding to the ‘caller action’ phase described in the Utstein-style [14]. Our findings align with international evidence on DA-CPR variability, where even minor wording differences can influence CPR performance [15]. These findings support our rationale for examining simplified, cognitively efficient instructions. Dispatcher instructions occur under high cognitive load, and simplification directly targets cognitive bottlenecks. Themes identified in our qualitative analysis—such as confusion with multi-step commands, difficulty maintaining attention, and emotional stress—mirror established barriers to telephone-assisted CPR reported in previous qualitative and mixed-methods studies, including caller distress, misinterpretation, and difficulty performing dispatcher instructions. This alignment reinforces the interpretation that cognitive and emotional overload substantially contributes to delayed or inconsistent bystander action. As this was a controlled simulation, caution is required when generalizing results to real-world dispatch settings, where emotional, logistical, and communication complexities are greater. The pattern of results faster recognition, quicker initiation, and lower perceived mental effort corresponds with predictions from CLT, suggestingthat streamlined instructions may reduce extraneous load and support task performance.

### Integration with CLT

Given that dispatcher instructions occur under high cognitive load requiring rapid interpretation, rapid action sequencing, and divided attention, the use of CLT-informed simplification directly addresses known cognitive bottlenecks in the dispatch-to-caller interaction [14]. According to CLT, task performance depends on the efficient allocation of limited working-memory resources among intrinsic, extraneous, and germane cognitive loads [15, 17–18]. When dispatcher instructions are verbose or fragmented, callers must devote excessive attention to decoding language rather than performing actions, thereby increasing extraneous load and reducing the cognitive capacity available for life-saving motor behavior. Simplifying and segmenting dispatcher prompts reduces this extraneous burden and enables germane processing, whereby cognitive resources are redirected toward schema formation and correct chest-compression execution.

This mechanism aligns with Paas et al., who demonstrated that learning efficiency improves when mental effort decreases while task performance is maintained—a relationship also evident in the lower perceived effort and faster recognition observed in the present study [13]. Furthermore, Sweller et al. expanded this framework, emphasizing that modern instructional design should minimize redundant processing, use clear signaling, and promote automaticity in task performance [17]. Applying these principles to dispatcher communication thus allows novice rescuers to focus on essential actions under stress, transforming a cognitively demanding script into an efficient, real-time instructional process.

From a design perspective, the ADDIE framework ensured that dispatcher message content was iteratively refined for clarity, sequencing, and motivational reinforcement. Integrating CLT with ADDIE enabled systematic reduction of redundant steps, prioritization of essential actions, and alignment of each instruction with cognitive processing limits. This approach likely enhanced situational comprehension and sustained caller engagement under pressure, as supported by qualitative feedback describing the simplified version as “easier to follow” and “less stressful.” Together, these findings highlight that optimizing cognitive load through instructional design is not only an educational consideration but also a potentially important operational factor in real-time emergency communication.

### Comparison with previous literature

Prior research has consistently shown that verbal complexity, fragmented instructions, and delayed task prompting hinder bystander performance during dispatcher-assisted resuscitation. Studies from Nordic and Central European dispatch systems report that variations in phrasing, questioning sequence, and speech tempo significantly influence the likelihood of bystander CPR and time to first compression [19]. Bathe et al. further demonstrated through a cluster-randomized simulation trial that optimizing dispatcher phrasing and delivery can reduce hesitation and improve basic life support (BLS) performance under dispatcher guidance [19].

Similarly, Dainty et al., Zhong et al. and Tuffley et al. demonstrated that emotional stress and fear of failure can suppress cognitive readiness, highlighting the need for emotionally attuned and cognitively efficient communication [7–8, 20]. The present study corroborates these insights, showing that simplified, confidence-oriented messages can offset stress-related cognitive interference among untrained callers.

In the Thai context, the NIEM 2013 DA-CPR protocol remains the national standard, but its complexity may inadvertently impose excessive cognitive load on lay callers. The simplified version used in this study, developed through iterative educational design, represents a pragmatic adaptation that preserves procedural accuracy while enhancing cognitive accessibility. These results suggest the potential value of evidence-informed dispatcher retraining aligned with human factors and instructional-design principles.

### Practical implications for EMS communication

Qualitative observations underscored the importance of spatial and postural guidance during dispatcher-assisted CPR. Novice rescuers frequently hesitated, misplaced hand position, or demonstrated suboptimal compression mechanics. Structured, spatially oriented cues—such as instructing callers to kneel beside the chest, keep the arms vertical, and pivot from the hips—appeared to support more consistent compression technique and posture. Continuous motivational feedback (e.g., “keep going,” “you’re doing well”) also helped sustain engagement and reduce premature cessation when dispatcher silence occurred. Taken together, these findings suggest that dispatcher communication functions as a dynamic learning interaction rather than a one-way command. Incorporating CLT-guided communication principles into dispatcher training may inform future training initiatives aligned with human factors and instructional design principles, particularly in educational and simulation-based contexts.

## Limitations

This single-center simulation with a modest sample of untrained university students limits generalizability; real-world callers are often older, emotionally distressed, or face environmental distractions that were absent in the study setting. The single-site academic sample *may* not reflect the psychological and situational stressors present in actual emergency calls. Although random assignment minimized selection bias, the lack of trial registration precludes classification as a formal randomized controlled trial.

Inter-rater reliability for video-based performance coding was not formally assessed, which may introduce minor observer bias in qualitative interpretation. Self-reported cognitive effort, while supported by established scales, remains subjective and may not fully capture real-time mental workload. Nevertheless, triangulation of quantitative and qualitative data strengthens confidence in the overall pattern of findings.

While this study supports theoretical and simulated improvements, integration with real-world dispatcher–caller data is needed. Previous national studies have reported similar challenges in cardiac arrest recognition and task initiation within Thailand’s EMS system, underscoring the importance of pragmatic evaluation in live dispatch environments.

## Future research

Future multicenter pragmatic studies should evaluate simplified scripts in actual dispatcher operations, linking communication process metrics to bystander and patient outcomes. Further research could integrate real dispatcher-caller audio recordings to assess the ecological validity and adaptability of simplified protocols across diverse populations, including those with limited language proficiency.

Objective measures of cognitive load—such as dual-task paradigms or eye-tracking—may complement self-reported scales and clarify the cognitive mechanisms underlying performance differences [13, 18].

Finally, exploration of AI-assisted, context-aware prompting systems could enable dispatchers to dynamically adjust phrasing based on caller behavior, potentially combining human empathy with adaptive instructional precision [21].

### Summary of key findings

Using a simplified dispatcher script was associated with earlier recognition and initiation of chest compressions, as well as lower perceived cognitive load among novice rescuers. These findings support a theory-driven, educational-design approach to optimizing dispatcher-assisted CPR communication.

## Conclusion

This simulation-based randomized study suggests that a CLT–ADDIE–informed dispatcher-assisted CPR script may improve selected time-critical performance metrics and reduce perceived cognitive load among untrained laypersons.

Simplified, action-oriented prompts and motivational cues were associated with improvements in the speed and accuracy of cardiac arrest recognition and compression initiation, while maintaining overall CPR quality.

These findings highlight the value of integrating instructional design principles into emergency communication systems. As dispatchers act as real-time educators, optimizing their verbal instruction through theory-informed frameworks **may support** bystander confidence, sustain engagement, and **facilitate more timely action**, with potential relevance to early components of the OHCA response pathway.

Future multicenter evaluations in live dispatch environments are warranted to validate these results and develop scalable retraining strategies within the NIEMS national dispatcher framework [9].

## Data Availability

All data generated or analyzed during this study are included in this published article.
